# Correction: Patch testing in Lao medical students

**DOI:** 10.1371/journal.pone.0236214

**Published:** 2020-07-09

**Authors:** Catriona I. Wootton, Mick Soukavong, Sonexai Kidoikhammouan, Bounthome Samountry, John S. C. English, Mayfong Mayxay

The sixth author's name is incorrect. The correct name is: Mayfong Mayxay. The correct citation is: Wootton CI, Soukavong M, Kidoikhammouan S, Samountry B, English JSC, Mayxay M (2020) Patch testing in Lao medical students. PLoS ONE 15(1): e0217192. https://doi.org/10.1371/journal.pone.0217192
