# Supplementary material for: Patch testing in Lao medical students
Source: PLoS One. 2020 Jan 16;15(1):e0217192. doi: 10.1371/journal.pone.0217192 (PMC6964850; doi:10.1371/journal.pone.0217192)
Supplement: S1 Table — (DOCX) [file pone.0217192.s001.docx]

S1 Table: TRUE Test® allergens

| **Allergen** | **µg/cm2** |
| --- | --- |
| Nickel sulphate | 200 |
| Wool alcohols | 1000 |
| Neomycin sulphate | 600 |
| Potassium dichromate | 54 |
| Caine mix | 630 |
| Fragrance mix | 430 |
| Colophony | 1200 |
| Paraben mix | 1000 |
| Balsam of Peru | 800 |
| Ethylenediamine dihydrochloride | 50 |
| Cobalt dichloride | 20 |
| p-tert-Butylphenol formaldehyde resin | 45 |
| Epoxy resin | 50 |
| Carba mix | 250 |
| Black rubber mix | 75 |
| Cl+ Me- Isothiazolinone | 4 |
| Quaternium-15 | 100 |
| Methyldibromo glutaronitrile | 5 |
| p-Phenylenediamine | 80 |
| Formaldehyde | 180 |
| Mercapto Mix | 75 |
| Thimerosal | 7 |
| Thiuram Mix | 27 |
| Diazolidinyl urea | 550 |
| Quinoline Mix | 190 |
| Tixocortol-21-pivalate | 3 |
| Gold Sodium Thiosulfate | 75 |
| Imidazolidinyl urea | 600 |
| Budesonide | 1 |
| Hydrocortisone-17-butyrate | 20 |
| Mercaptobenzothiazole | 75 |
| Bacitracin | 600 |
| Parthenolide | 3 |
| Disperse blue 106 | 50 |
| 2-Bromo-2-nitropropane-1,3-diol (Bronopol) | 250 |
